# Engineering stem cells to produce exosomes with enhanced bone regeneration effects: an alternative strategy for gene therapy

**DOI:** 10.1186/s12951-022-01347-3

**Published:** 2022-03-15

**Authors:** Feiyang Li, Jun Wu, Daiye Li, Liuzhi Hao, Yanqun Li, Dan Yi, Kelvin W. K. Yeung, Di Chen, William W. Lu, Haobo Pan, Tak Man Wong, Xiaoli Zhao

**Affiliations:** 1 Research Center for Human Tissue and Organs Degeneration, Institute of Biomedicine and Biotechnology, Shenzhen Institute of Advanced Technology, Chinese Academy of Sciences, Shenzhen, 518055 China; 2 Shenzhen Key Laboratory for Innovative Technology in Orthopaedic Trauma, The University of Hong Kong-Shenzhen Hospital, Shenzhen, 518053 China; 3 University of Chinese Academy of Sciences, Beijing, 100049 China; 4 Department of Orthopaedics and Traumatology, The University of Hong Kong, Hong Kong, 999077 China

**Keywords:** Exosomes, Stem cell, Gene therapy, Tissue regeneration, Cell-free therapy

## Abstract

**Background:**

Exosomes derived from stem cells have been widely studied for promoting regeneration and reconstruction of multiple tissues as “cell-free” therapies. However, the applications of exosomes have been hindered by limited sources and insufficient therapeutic potency.

**Results:**

In this study, a stem cell-mediated gene therapy strategy is developed in which mediator mesenchymal stem cells are genetically engineered by bone morphogenetic protein-2 gene to produce exosomes (MSC-BMP2-Exo) with enhanced bone regeneration potency. This effect is attributed to the synergistic effect of the content derived from MSCs and the up-regulated *BMP2* gene expression. The MSC-BMP2-Exo also present homing ability to the injured site. The toxic effect of genetical transfection vehicles is borne by mediator MSCs, while the produced exosomes exhibit excellent biocompatibility. In addition, by plasmid tracking, it is interesting to find a portion of plasmid DNA can be encapsulated by exosomes and delivered to recipient cells.

**Conclusions:**

In this strategy, engineered MSCs function as cellular factories, which effectively produce exosomes with designed and enhanced therapeutic effects. The accelerating effect in bone healing and the good biocompatibility suggest the potential clinical application of this strategy.

**Graphical Abstract:**

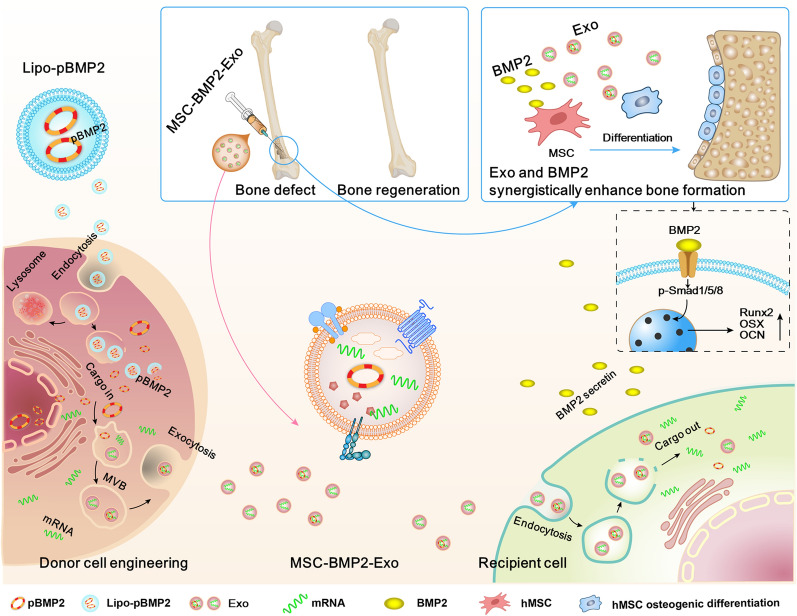

**Supplementary Information:**

The online version contains supplementary material available at 10.1186/s12951-022-01347-3.

## Introduction

The beneficial effects of mesenchymal stem cell-derived exosomes (MSC-Exo) in tissue regeneration have attracted significant interest in their applications in cell-free therapies [1]. Growing evidence has revealed that transplanted MSCs function rely on their paracrine actions through MSC-Exo in repairing injured tissue [2]. The positive roles of MSC-Exo have been proven in the repair and reconstruction of multiple tissues, including cartilage, skin, and skeleton [3–6]. Allogeneic MSC-Exo-based therapeutics are currently being assessed by early phase clinical trials in regenerative and anti-inflammatory applications [7]. Therefore, MSC-Exo-based therapeutics provide a promising strategy for skeletal tissue regeneration to meet the increasing demands raised by nonunion fractures, severe trauma, malignancy resection, and aging of the population [8]. However, the regeneration capacities of exosomes are susceptible to MSC sources, as well as cell status, and further improvements are usually required [9].

Exosomes elicit biological functions and regulate the cellular responses of recipient cells by transporting cargos involved in various biological processes, even over long distances [10]. The content of exosomes, including proteins, RNA, lipids, DNA, metabolites, and carbohydrates, varies with the type and status of the donor cells [11]. Therefore, exosomes can reflect the phenotype of donor cells and can even present a specific therapeutic function [12]. Exosomes derived from neonatal human umbilical cord MSCs have shown rejuvenating effects on adult stem cell, which provides a new strategy for degenerative bone disease [13]. Compared with cell-based therapy, MSC-Exo can modulate phenotypes of recipient cells in a safe and efficient way without immunological or ethical restrictions [14, 15]. However, the mechanisms underlying the packaging of cargos into exosomes in cells have not been clearly elucidated [16, 17]. Therefore, how to acquire exosomes loaded with desired cargos for specific therapeutic effects and to preserve the beneficial features of exosomes as much as possible is the major challenge for the clinical applications of exosomes.

Several studies have focused on engineering exosomes, either by directly modifying exosomes or by regulating the cellular response of donor cells with certain biocues, to enhance their therapeutic effects [18–20]. Regulating the content of exosomes by manipulating their donor cells is relatively simple and has no adverse effect on the structural integrity of exosomes, which facilitates the maintenance of their function [21]. Upon osteogenic induction, MSC-Exos show enhanced osteoinductive potential [22]. The altered culture conditions change the genetic information carried by the exosomes [8]. Enhancing a specific gene expression in MSCs upregulates the corresponding cargos of exosomes to induce prolonged therapeutic effects [23]. Therefore, exosomes are being actively investigated as a novel shuttle for the delivery of therapeutic nucleic acid across biological barriers [24].

Exosomes are naturally occurring membrane-enclosed nano-sized vesicles approximately 40–150 nm in diameter, secreted by most living cells through fusion of multivesicular bodies (MVBs) with the plasma membrane [25]. They resemble liposomes in terms of size, shape, and structure. Liposome have been widely used as non-viral vehicles for gene delivery, whereas, exosomes provide highly complex bilayers, containing up to hundreds of different lipid, protein, and carbohydrate types, as well as internal cargos and surface-associated molecules [26]. These complex constituents protect the internal cargos from degradation and prolong systemic retention with minimum adverse effects, which are the main obstacles faced by synthetic nano formulations in clinical translation [27–29]. Extensive studies have modified synthesized nanoparticles with multiple functional components to mimic biological structures for improving delivery efficiency, however, this process seems impractical and incompatible with large-scale clinical-grade manufacturing [26]. Furthermore, exosomes are biocompatible with the host immune system and have the innate ability to cross biological barriers in vivo [30]. They are well tolerated when administrated through repeated injection in mice [31]. In addition, their small size and deformable cytoskeleton allow them to penetrate deep tissues [32]. Especially, MSC-Exos have shown homing ability to the injured tissue [33]. These features have made exosomes more effective and efficient than synthetic nanocarriers in nucleic acid delivery for tissue regeneration.

Therefore, exosome-mediated bone regeneration can be achieved by modulating the cargos carried by the exosomes through genetically engineering the mediator MSCs. In this study, human fetal bone marrow derived mesenchymal stem cells (hMSCs) were induced to produce exosomes (MSC-BMP2-Exo) with osteogenic effects by upregulating bone morphogenetic protein-2 (*BMP2*) gene expression through liposome-mediated gene transfection. *BMP2* gene was used as a model to demonstrate the feasibility of this strategy for tissue regeneration, and this could be applied to other cellular regulatory factors. MSC-BMP2-Exo significantly promoted bone healing in mouse bone defect model. The bone healing capacities of MSC-BMP2-Exo are attributed to the synergistic effect of the content derived from stem cells and the up-regulated mRNA expression of osteogenesis-related genes. Hence, in this strategy, mediator stem cells function as cellular factories, which effectively produce exosomes with designed and enhanced therapeutic effects. Importantly, the toxic effects of liposomes for *BMP2* gene transfection on donor stem cells are borne by these cellular factories, while their exosomes exhibit excellent biocompatibility and bioavailability. Comparing with direct exosomal engineering and utilizing viral vector, this strategy has advantage of biosafety, which could facilitate the clinical translation to promote tissue repair [22]. The accelerating effect in bone defect healing and good biocompatibility suggests the potential clinical application of this strategy. In addition, by plasmid tracking, it is interesting to find a portion of plasmid DNA was encapsulated by exosomes and delivered to recipient cells. By inhibiting lysosomal-dependent degradation, the amount of plasmids carried by exosomes is increased. This technology may be applied in the production of exosome-based gene transfection vehicles. This study delivers the concept of combining exosome-mediated therapies and gene delivery technologies, MSCs may become pharmaceutical factories producing therapeutic exosomes.

## Results

### Genetically engineered hMSCs by *BMP2* gene delivery alter exosomal content

**Fig.1:**
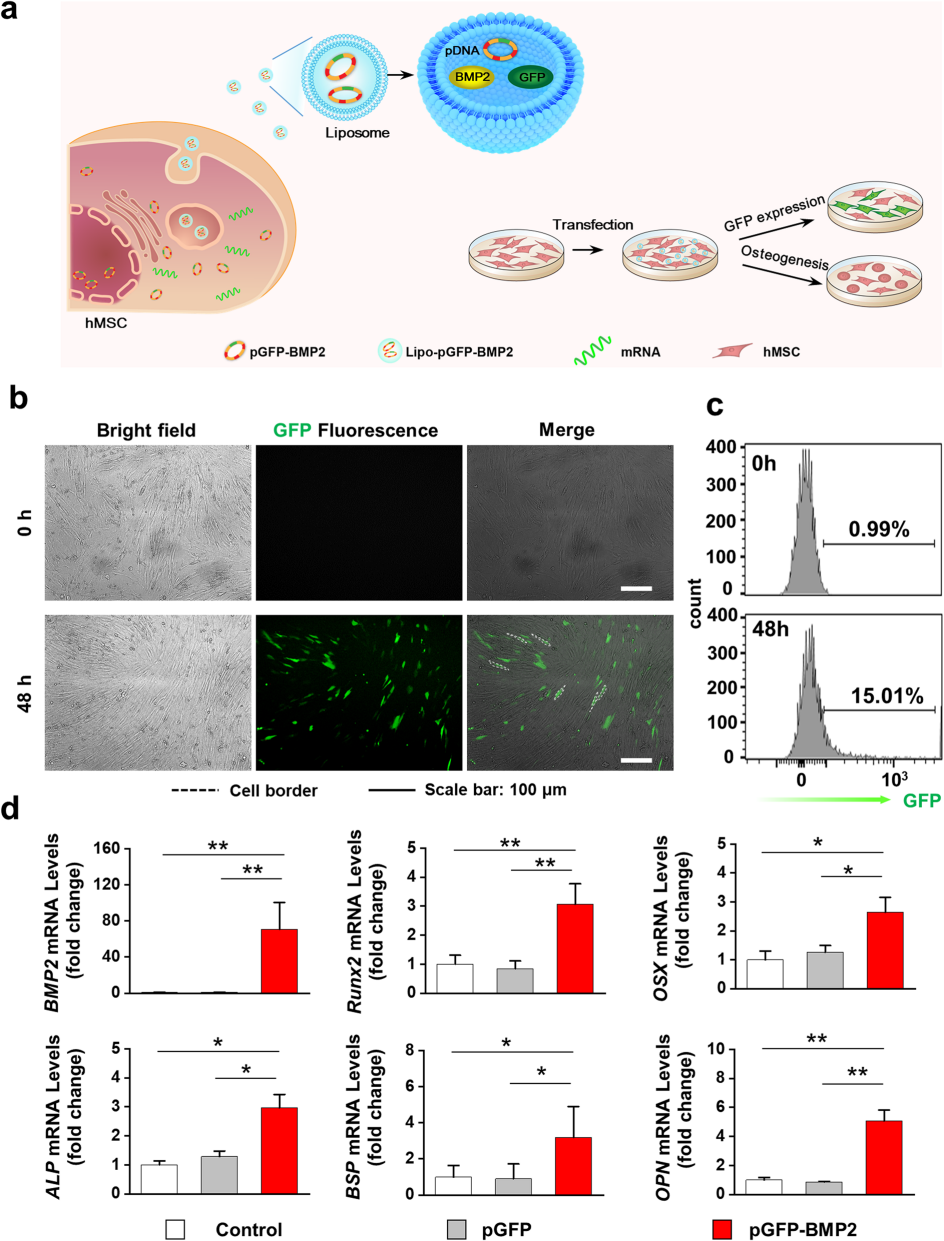
HMSCs were genetically engineered by upregulating *BMP2* gene expression. **a** A schematic illustration of genetically engineering hMSCs by liposome mediated *BMP2* gene delivery. **b**, **c** Fluorescent images of hMSCs transfected by liposome/pGFP-BMP2 plasmid, and transfection efficiency was analyzed by flow cytometry, Scale bar: 100 μm. **d** mRNA expressions of osteogenic differentiation-related markers (*BMP2*, *Runx2*, *OSX*, *ALP*, *BSP*, *OPN*) in hMSCs were analyzed by qRT-PCR after transfection. Untransfected hMSCs were detected as control, n = 3. *p < 0.05, **p < 0.01

Human fetal bone marrow derived mesenchymal stem cells (hMSCs) were engineered by *BMP2* gene transfection to generate exosomes with altered profiles and cargos to promote the bone regeneration effect of MSC-Exo. The *BMP2* gene was delivered into hMSCs by using liposome (Lipofectamine 3000) (Fig. 1a). The plasmid DNA (pGFP-BMP2) encodes both *BMP2* and green fluorescent protein (*GFP*) to facilitate the evaluation of transfection efficiency. Green fluorescence illuminated by hMSCs after transfection demonstrated the successful expression of plasmid DNA (Fig. 1b). The transfection efficiency was 15.01%, as quantified by flow cytometry (Fig. 1c). The mRNA expression of *BMP2* in hMSCs were significantly up-regulated after transfection, which suggested the expression of the *BMP2* gene in hMSCs. The osteogenic-related genes such as runt-related transcript factor 2 (*Runx2*), osterix (*OSX*), alkaline phosphate (*ALP*), bone sialoprotein (*BSP*), and osteopontin (*OPN*), were also enhanced (Fig. 1d). By contrast, hMSCs transfected with pGFP showed results similar to those in the untransfected control group. Furthermore, the expressed BMP2 protein level in pGFP-BMP2 transfected group was also significantly higher than those in control and pGFP groups (Additional file 1: Fig. S1). These results indicated that *BMP2* gene delivery initiated the osteogenic differentiation of hMSCs.

**Fig. 2:**
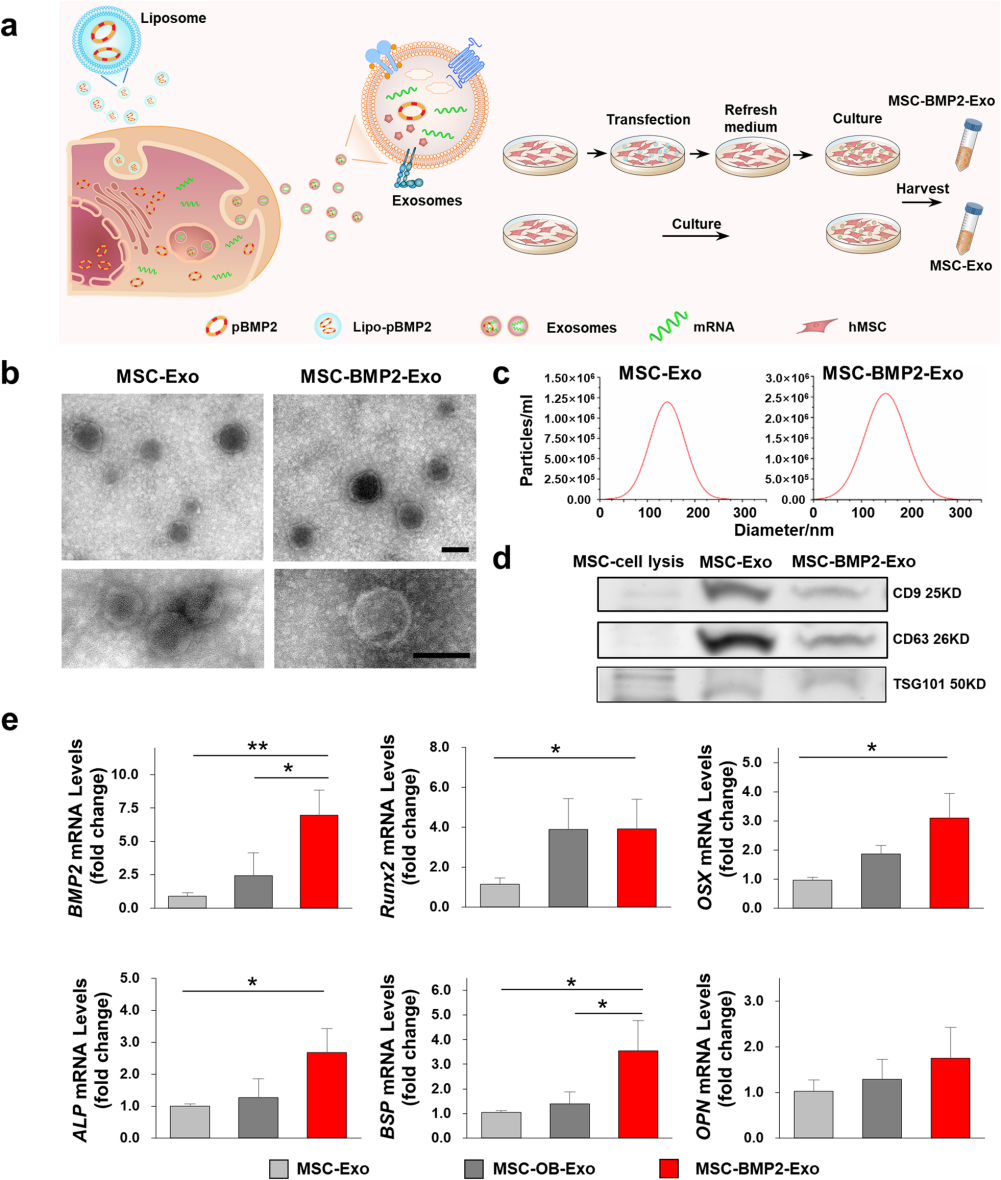
*BMP2* gene engineering altered the internal mRNA content of derived exosomes. **a** A schematic illustration of exosomes harvest after hMSCs genetically engineering. **b** The morphology of MSC-Exo and MSC-BMP2-Exo was observed by TEM. Scale bar: 100 nm. **c** Size distributions of exosomes were determined by nanoparticle tracking analysis. **d** Exosomal markers CD63, CD9 and TSG101 in cell and exosome lysates were detected by western blot. **e** The internal mRNA expressions of osteogenic differentiation-related genes in MSC-Exo, MSC-OB-Exo and MSC-BMP2-Exo were analyzed by qRT-PCR, including *BMP2*, *Runx2*, *OSX*, *ALP*, *BSP* and *OPN*, n = 3. *p < 0.05, **p < 0.01

The exosomes secreted from hMSCs (MSC-Exo) and *BMP2* genetically engineered hMSCs (MSC-BMP2-Exo) were harvested and investigated (Fig. 2a). These exosomes appeared to be spherical nanoparticles in TEM images and presented similar diameters at approximately 150 nm, as determined by nanoparticle tracking analysis (NTA) (Fig. 2b, c). Their zeta potential were around − 5 mV, and slightly raised to − 4 mV after 3 days. There is no difference between MSC-Exo and MSC-BMP2-Exo (Additional file 1: Fig. S2). Classical exosomal markers were characterized by Western blot analysis. Both the MSC-Exo and MSC-BMP2-Exo were positive for membrane markers CD9 and CD63 and proteins related to the ESCRT-independent pathway TSG101 (Fig. 2d). These results demonstrated that the nanoparticles extracted from hMSCs were mainly exosomes. The internal nucleic acid contents of exosomes were analyzed. Interestingly, mRNA expressions of osteogenic differentiation-related genes in exosomes, including *BMP2*, *Runx2*, *OSX*, *ALP*, *BSP*, and *OPN*, were significantly up-regulated in MSC-BMP2-Exo compared with those in MSC-Exo. These were also higher than those in exosomes derived from osteogenic-induced hMSCs (MSC-OB-Exo) (Fig. 2e), suggesting mRNA content of exosomes could be modulated by engineering their donor cells.

### MSC-BMP2-Exo can safely and effectively promote osteogenic differentiation in vitro.

**Fig. 3:**
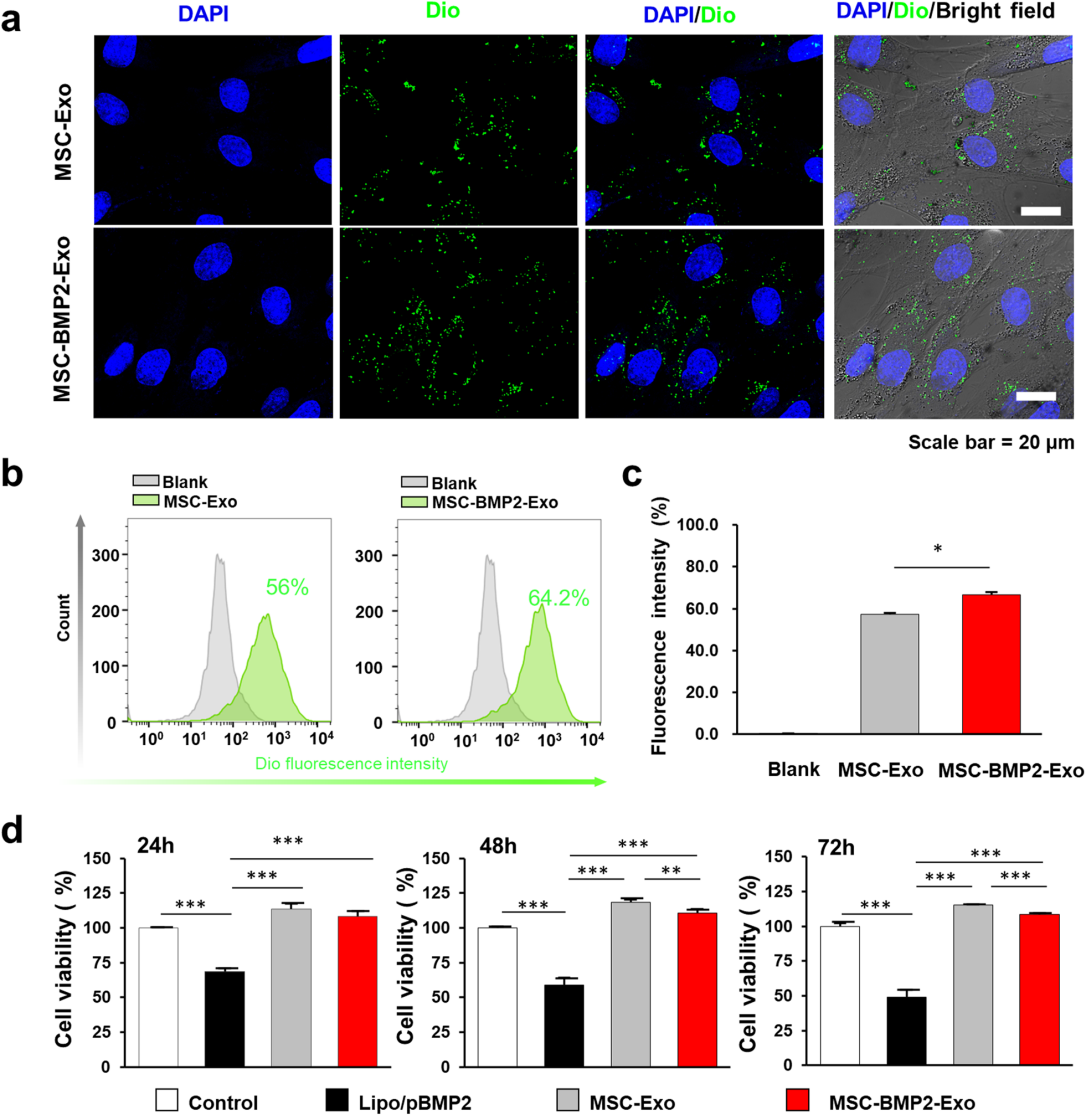
MSC-BMP2-Exo exhibited excellent cellular uptake efficiency and biocompatibility. **a** Fluorescent microscopic image showed the cellular internalization of MSC-BMP2-Exo and MSC-Exo by recipient cells after overnight incubation. Exosomes were stained by Dio with green fluorescent, Scale bar: 20 μm. **b** Flow cytometry analysis of Dio-labeled exosomes engulfed by recipient cells. **c** Ratios of recipient cells that had engulfed MSC-BMP2-Exo or MSC-Exo. Cells cultured in normal medium without exosomes were also detected as control group, n = 3. **d** Cell viability of hMSCs incubated with exosomes and Lipo/pBMP2 complex was determined by CCK-8 assay, n = 6. *p < 0.05, **p < 0.01, ***p < 0.001

Safety and therapeutic effects are essential in promoting the application of exosome-based therapy. Both MSC-Exo and MSC-BMP2-Exo could be uptaken by recipient MSC cells. Through fluorescent staining with 3,3’-dioctadecyloxacarbocyanine perchlorate (Dio), exosomes exhibiting green fluorescence could be found in the cytoplasm of recipient cells after overnight incubation (Fig. 3a). Cellular uptake was further quantified by flow cytometry. The percentage of recipient cells that had engulfed exosomes was approximately 56.0% and 64.2% for MSC-Exo and MSC-BMP2-Exo, respectively (Fig. 3b, c). These results demonstrated that both MSC-Exo and MSC-BMP2-Exo had excellent cellular uptake efficiency. The higher cellular uptake efficiency for MSC-BMP2-Exo may be because the engineered MSC-BMP2-Exos increase the binding and interaction between exosomes and hMSCs, making it easier to be taken up by target cells [34]. As a therapeutic gene delivery vehicle, the biocompatibility of exosomes was evaluated. Both exosomes exhibited no toxic effect on hMSCs after 72 h of incubation (Fig. 3d). The slightly lower cell proliferation in MSC-BMP2-Exo group compared with MSC-Exo group may be associated with their different cellular uptake efficiency. However, the complex of liposome/pBMP2 plasmid was incubated with cells in a recommended concentration and resulted in cell viability lower than 50%. This result suggested that even though mediator cells bear the inherent toxic effects of gene delivery vehicles, their cargo-carrying exosomes will not transfer these effects to recipient cells.

**Fig. 4:**
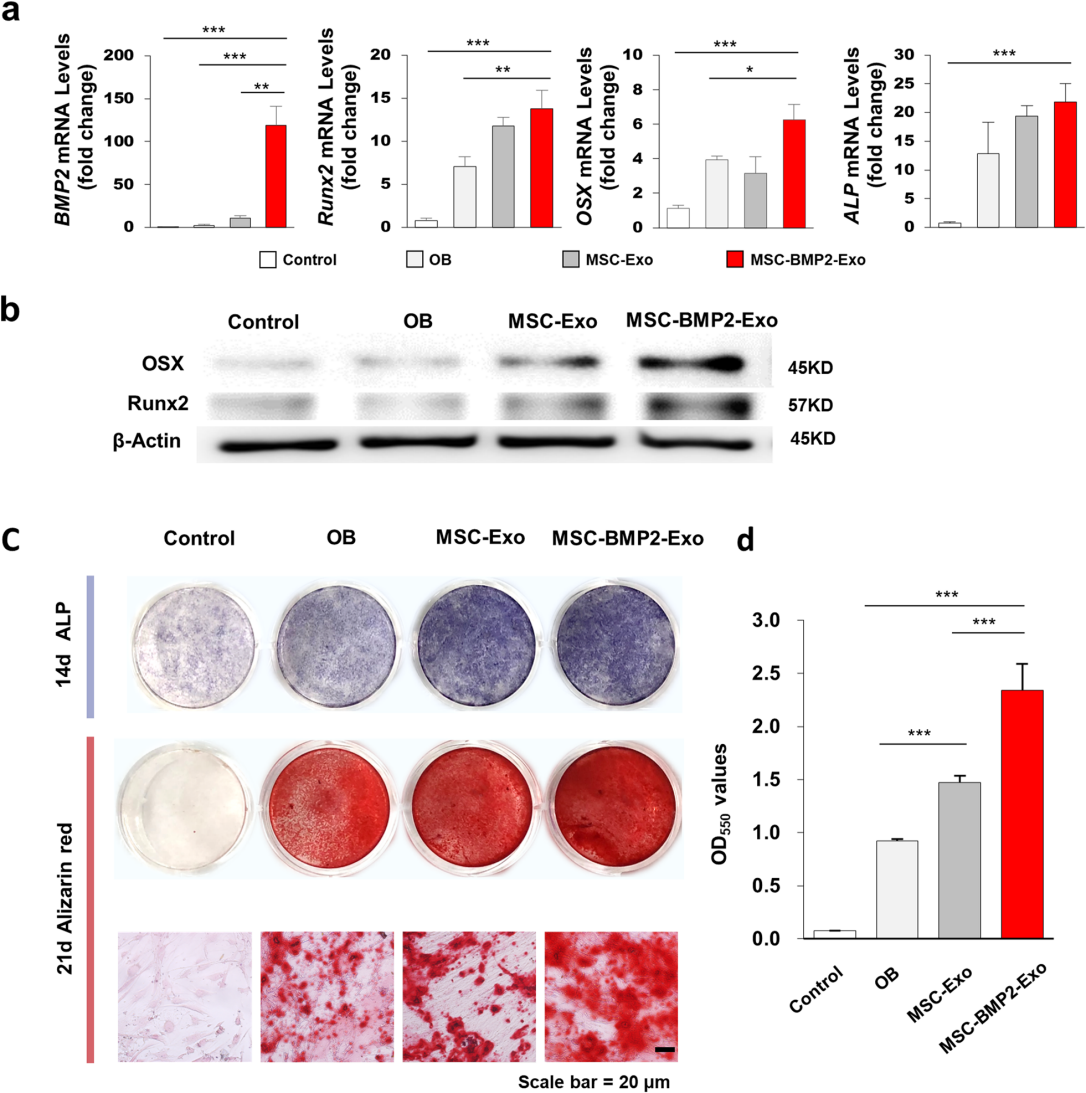
MSC-BMP2-Exo promoted osteogenic differentiation of recipient hMSCs in vitro. **a** Osteogenic differentiation-related mRNA expressions of hMSCs, including *BMP2*, *Runx2*, *OSX* and *ALP*, were analyzed by qRT-PCR after 5 days of culture, n = 3. Cells were cultured in normal culture medium (control group), differentiation medium (OB group), differentiation medium supplemented with MSC-Exo (MSC-Exo group), and differentiation medium supplemented with MSC-BMP2-Exo (MSC-BMP2-Exo group). **b** Western blot analysis of recipient cells on the protein expression of Runx2 and OSX after 7 days of culture. Loading control was β-actin. **c** ALP and alizarin red staining of hMSCs under different treatment after 14 and 21 days of culture. Upper graph: ALP staining, lower graph: alizarin red staining. **d** Alizarin red staining was quantified by dissolving calcium nodules with 10% hexadecylpyridinium chloride monohydrate solution, n = 3. *p < 0.05, **p < 0.01, ***p < 0.001

The osteogenic promotion effects of exosomes were investigated in vitro. Osteogenic induction promoted osteoblast lineage commitment, and the related gene expressions, including *Runx2*, *OSX* and *ALP*, were up-regulated, as observed in OB group after 5 days of treatment (Fig. 4a). MSC-BMP2-Exo supplied in differentiation medium could significantly upregulate the expression of *BMP2* gene expression and thus further promoted *Runx2* and *OSX* expressions. Western blot analysis showed that the protein levels of OSX and Runx2 were significantly increased in the MSC-BMP2-Exo group at day 7 (Fig. 4b). The enhanced osteogenic effect of MSC-BMP2-Exo on hMSCs was further confirmed by ALP and Alizarin red staining (Fig. 4c). Alizarin red staining and quantitative analysis revealed that MSC-BMP2-Exo could increase calcium nodule formation at day 21 (Fig. 4c, d). These results suggested the effect of MSC-BMP2-Exo on modulating the osteogenic functions of recipient hMSCs.

### MSC-BMP2-Exo expedites regeneration in both trabecular and cortical bones through BMP2/Smad pathway.

**Fig. 5:**
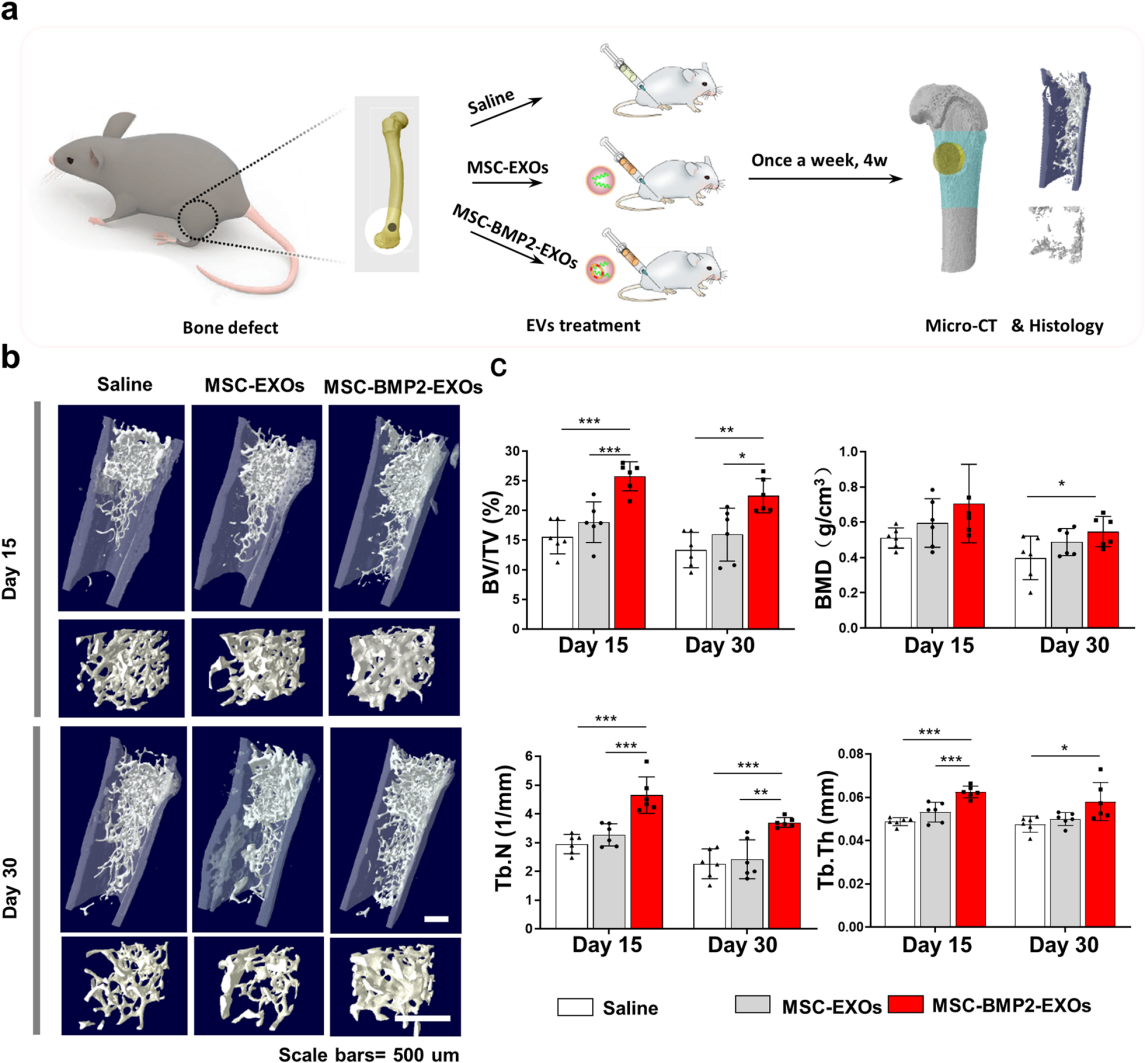
MSC-BMP2-Exos expedited bone regeneration through BMP2/Smad pathway. **a** 8-week-old male C57BL/6 mice were used for animal study. Holes with diameters of 1 mm were created at lateral epicondyle of right distal femur. At each time point, micro-CT, histology and immunohistochemistry analyses were performed. **b** Representative reconstructed 3D images of trabecular bone at injured sites. Scale bars: 500 μm. **c** Quantitative analysis of BMD, BV/TV, Tb.Th and Tb.N for trabecular bones at injured sites, n = 6. *p < 0.05, **p < 0.01, ***p < 0.001

**Fig. 6:**
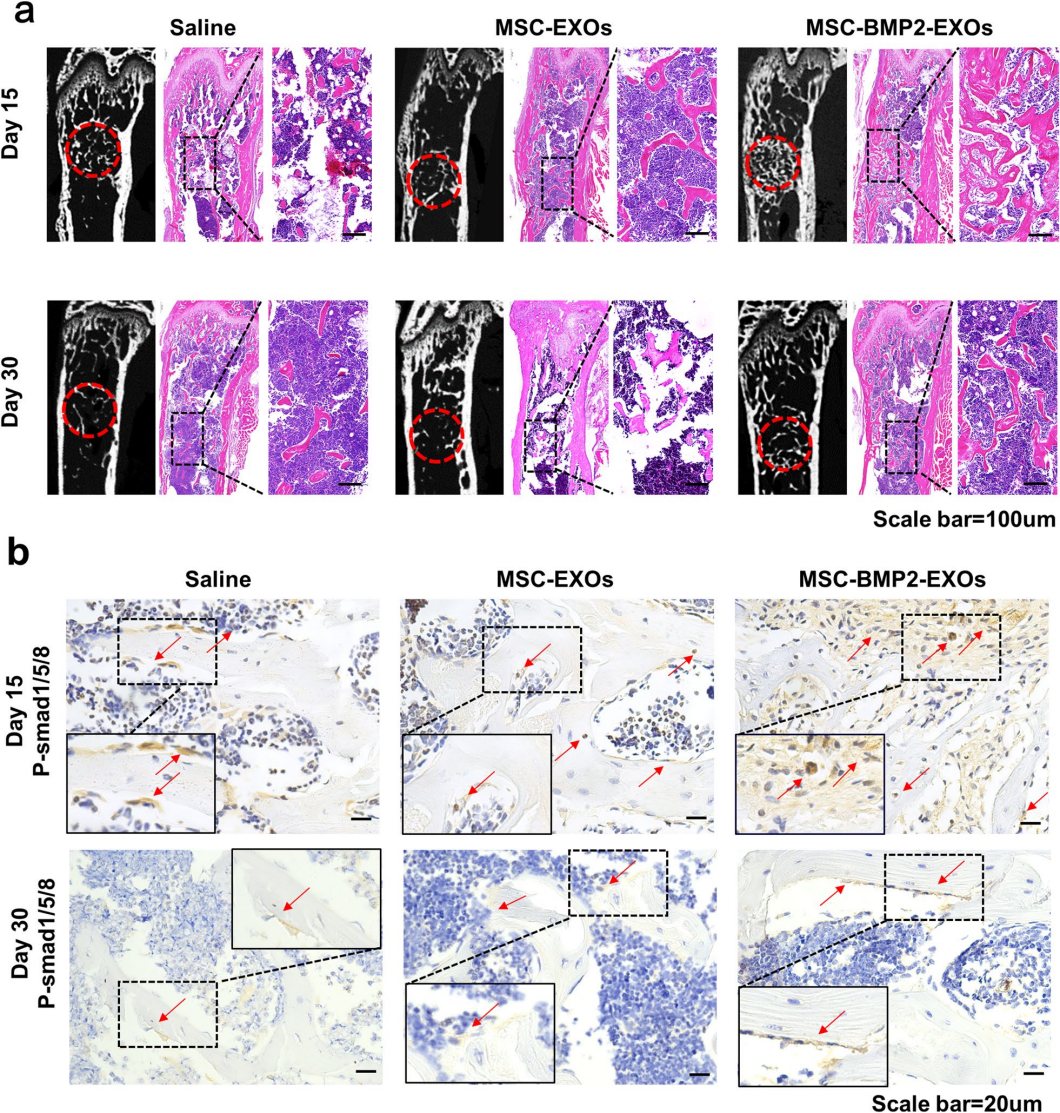
Immunohistochemical analysis of BMP2/Smad pathway. **a** Radiographic images and H&E staining of mice distal femur. The injured areas are highlighted in red or black dotted lines. Scale bars: 200 μm. **b** Immunohistochemical staining of Smad1/5/8 marker at injured sites of trabecular bone. Red arrows indicate Smad1/5/8 positive cells. Scale bar: 20 μm

The effect of MSC-BMP2-Exo on bone regeneration was further evaluated in vivo. Whether MSC-BMP2-Exo could expedite bone healing was investigated with a mouse model with lateral epicondyle defects at the distal femur (Fig. 5a). Thirty-six mice with femoral defects were divided into three groups, which were treated with either 0.9% saline, MSC-Exo, or MSC-BMP2-Exo, with twelve mice in each group. Exosomes or saline was injected once a week. The bone regeneration effects were investigated and analyzed by micro-computed tomography (micro-CT) (Fig. 5b, c). Reconstructed three-dimensional images and structural parameters of the femur suggested that MSC-BMP2-Exo increased trabecular bone mass. The bone volume fraction (BV/TV) and trabecular bone number (Tb.Th) were significantly increased in MSC-BMP2-Exo group at day 15 compared with those in mice injected with saline or MSC-Exo. H&E staining also proved that the MSC-BMP2-Exo group exhibited more new bone formations (Fig. 6a). The decrease of BV/TV on day 30 compared with that on day 15 suggested the bone remodeling process after defect healing. Immunohistochemical assay of Smad1/5/8, the downstream markers of BMP2, revealed that many Smad1/5/8 positive cells were observed at day 15 around the injured sites in the MSC-BMP2-Exo treated group (Fig. 6b). This finding proved that the MSC-BMP2-Exo improved trabecular bone regeneration through the BMP2/Smad signaling pathway.

**Fig. 7:**
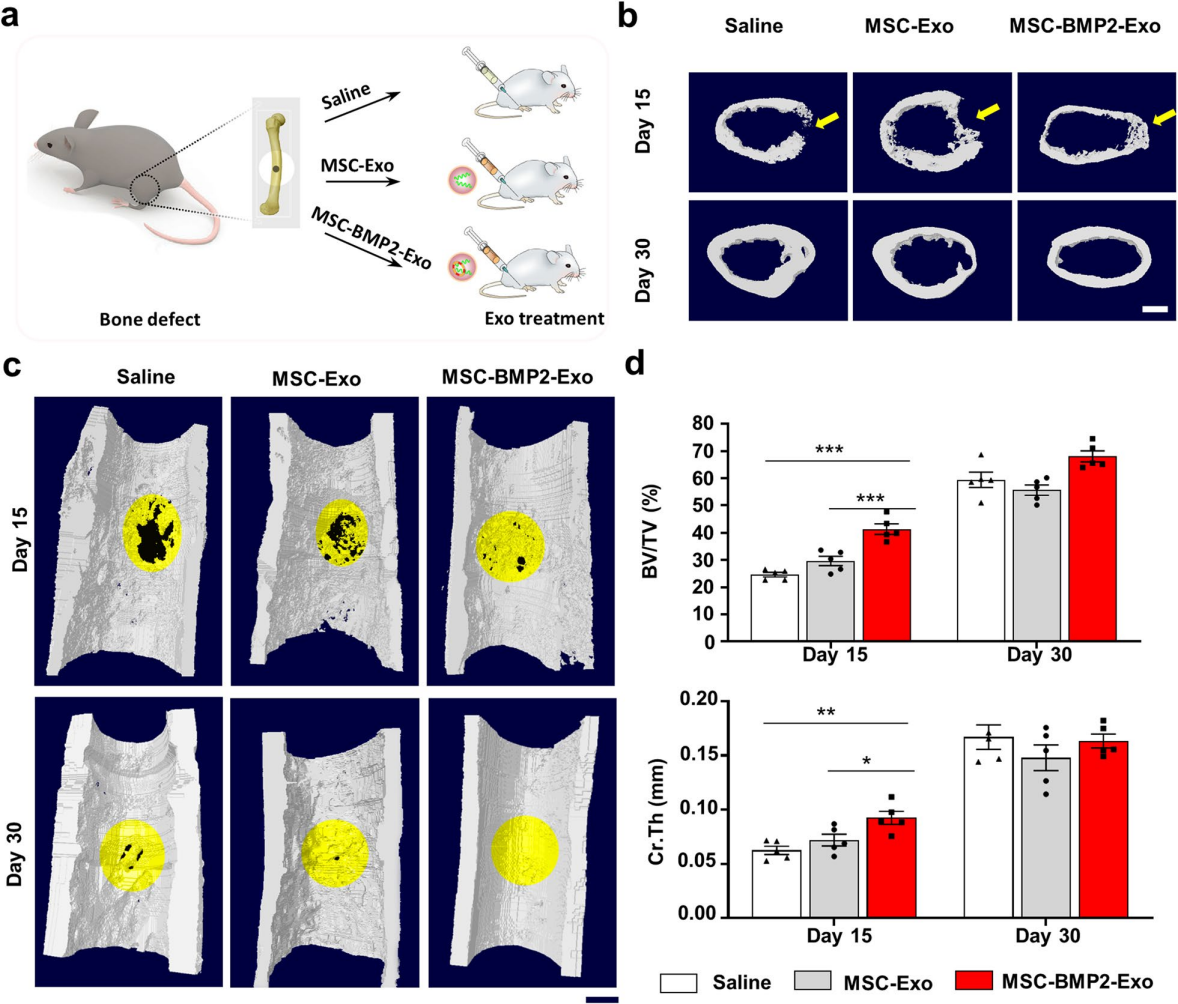
MSC-BMP2-Exos expedited cortical bone regeneration. **a** Holes with diameters of 1 mm were created at mid-diaphysis of femora. Micro-CT was performed on day 15 and day 30. **b**, **c** Representative reconstructed 3D images of cortical bone at injured sites. The injured areas are highlighted by yellow color. Scale bars: 500 μm. **d** Quantitative analysis of BV/TV and Cr.Th for cortical bones at injured sites, n = 5. *p < 0.05, **p < 0.01, ***p < 0.001

The effect of MSC-BMP2-Exo on cortical bone regeneration was further investigate. Fifteen mice with femoral defects at the mid-diaphysis of the femur were divided into three groups. Each group was treated with either 0.9% saline, MSC-Exo, or MSC-BMP2-Exo (Fig. 7a). Micro computed tomography (micro-CT) indicated that the weekly local injection of MSC-BMP2-Exo significantly enhanced the regeneration of cortical bone at the injured sites (Fig. 7b). The reconstructed three-dimensional images showed that the MSC-BMP2-Exo group significantly promoted the healing of defects compared with that in the control and MSC-Exo groups at day 15 (Fig. 7c). The structural parameters of the BV/TV and cortical thickness (Cr.Th) at the injured sites also showed a corresponding tendency (Fig. 7d).

### In vivo distribution of exosomes after local and systemic injections

**Fig. 8:**
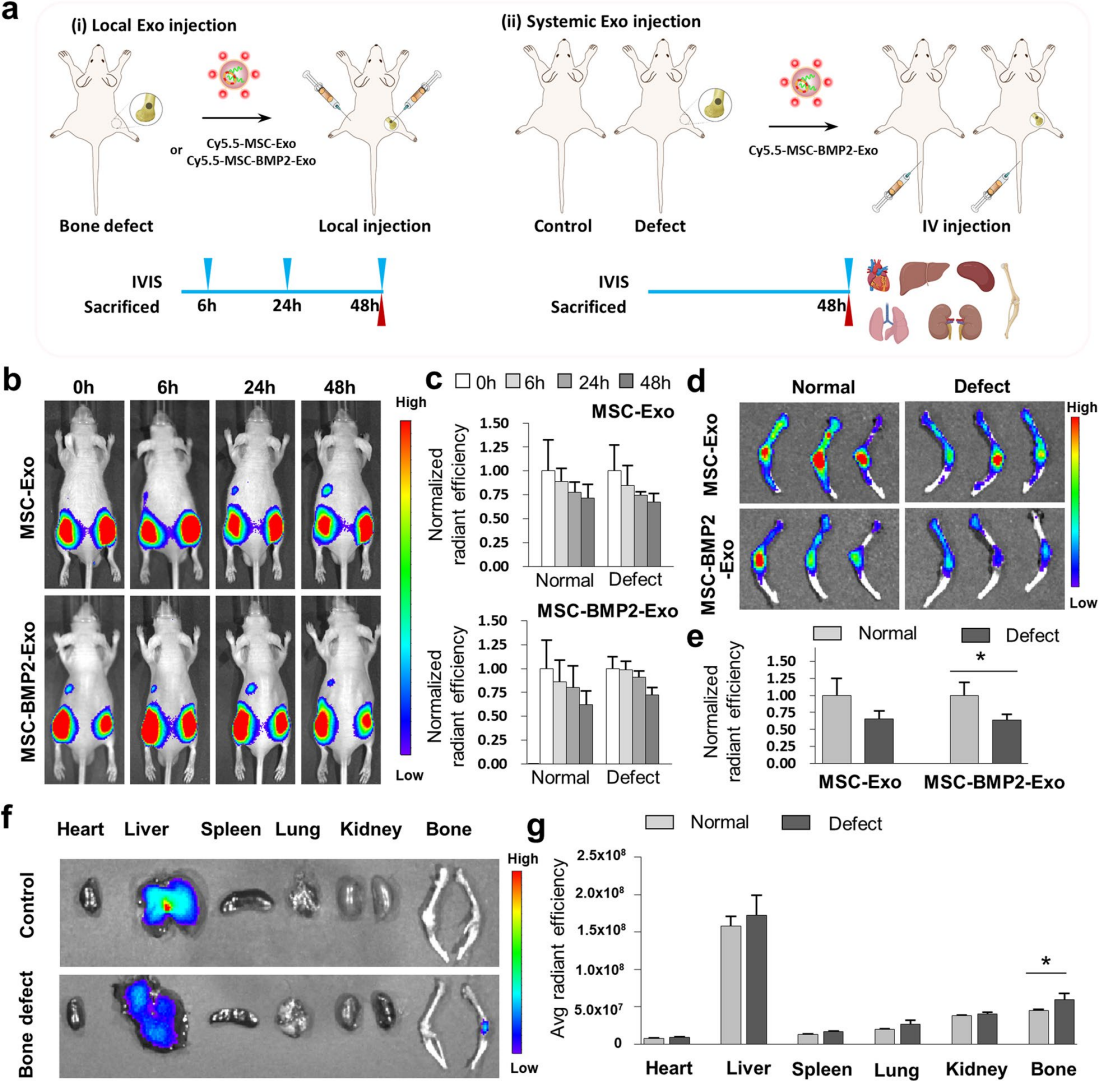
In vivo retention and distribution of exosomes after local and systemic injection. **a** Nude mice with bone defects on right distal femurs were created. (i) For exosomes retention, Cy5.5 labelled exosomes were treated by local injection on both sides, and the retention of exosomes was imaged by IVIS Imaging System at 6, 24 and 48 h. (ii) For exosome distribution, Cy5.5 labelled exosomes were treated by intravenously injected through the tail vein, and distribution of exosomes in different organs was imaged at 48 h. **b**, **c** The retention of exosomes was imaged after local injection and performed quantitative analysis of fluorescent intensities at injection sites, n = 3. **d**, **e** Femurs and tibias of both sides in MSC-BMP2-Exo and MSC-Exo groups were excised at 48 h after exosome injection visualized by biofluorescence imaging, and performed quantitative analysis of fluorescent intensities at injection sites, n = 3. **f**, **g** Biodistribution of Cy5.5 labelled MSC-BMP2-Exo in major organs (heart, liver, spleen, lung, kidney, and femur) was visualized at 48 h after intravenously injection, and performed quantitative analysis of fluorescent intensities, n = 3. Bone defect was made on right distal femurs, and mice without bone defect was compared. *p < 0.05, **p < 0.01, ***p < 0.001

Given that exosomes were directly injected at the bone injury sites, the retention and distribution of exosomes in tissues were closely related to the regeneration effect. Bone defects were created at the right sides of the distal femur on nude mice, whereas the left sides were left intact, followed by local injection of Cy5.5 labeled exosomes on both sides (Fig. 8a[i]). By using in vivo fluorescence imaging, the intensities of both MSC-Exo and MSC-BMP2-Exo could be retained at the injection sites for more than 48 h, no matter a bone defect was present or not (Fig. 8b, c). The femora were then excised and imaged, and the fluorescence intensity was still be observed, indicating the retention of exosomes at the bone tissue (Fig. 8d, e). In the defect group, exosomes were directly injected into the bone marrow through the created bone defect, therefore the decreased fluorescence intensity was observed for the release of exosomes from bone marrow into systemic circulation. The shielding of bone tissue to the fluorescence may also influence the intensity. These results suggested that exosomes could be retained at bone injured sites for several days to fulfill the therapeutic function after local injection.

The retention of exosomes in bone marrow may be related to the homing effect to the injured tissue. Therefore, whether the injured tissue would influence the distribution of exosomes was then studied through systemic injection. Mice with bone defects at the right femur (defect group) were compared with mice without any bone defect (control group), and Cy5.5-labeled exosomes were intravenously injected into tail vein (Fig. 8a[ii]). The distribution of exosomes in different organs was investigated 48 h after injection. Little residual fluorescence was detected in heart, spleen, lung, and kidney of both groups, while strong fluorescence was observed in liver of both groups with no significant difference. MSC-Exo and MSC-BMP2-Exo showed no cytotoxicity to hepatocytes (Additional file 1: Fig. S3). Interestingly, the injured femur showed higher fluorescence intensity than that in the control group (Fig. 8f, g), which might suggest that the injured bone attracted the homing of MSC derived-exosomes.

### Plasmid DNAs can be packaged by donor cells and transported to recipient cells through secreted Exosomes

**Fig. 9:**
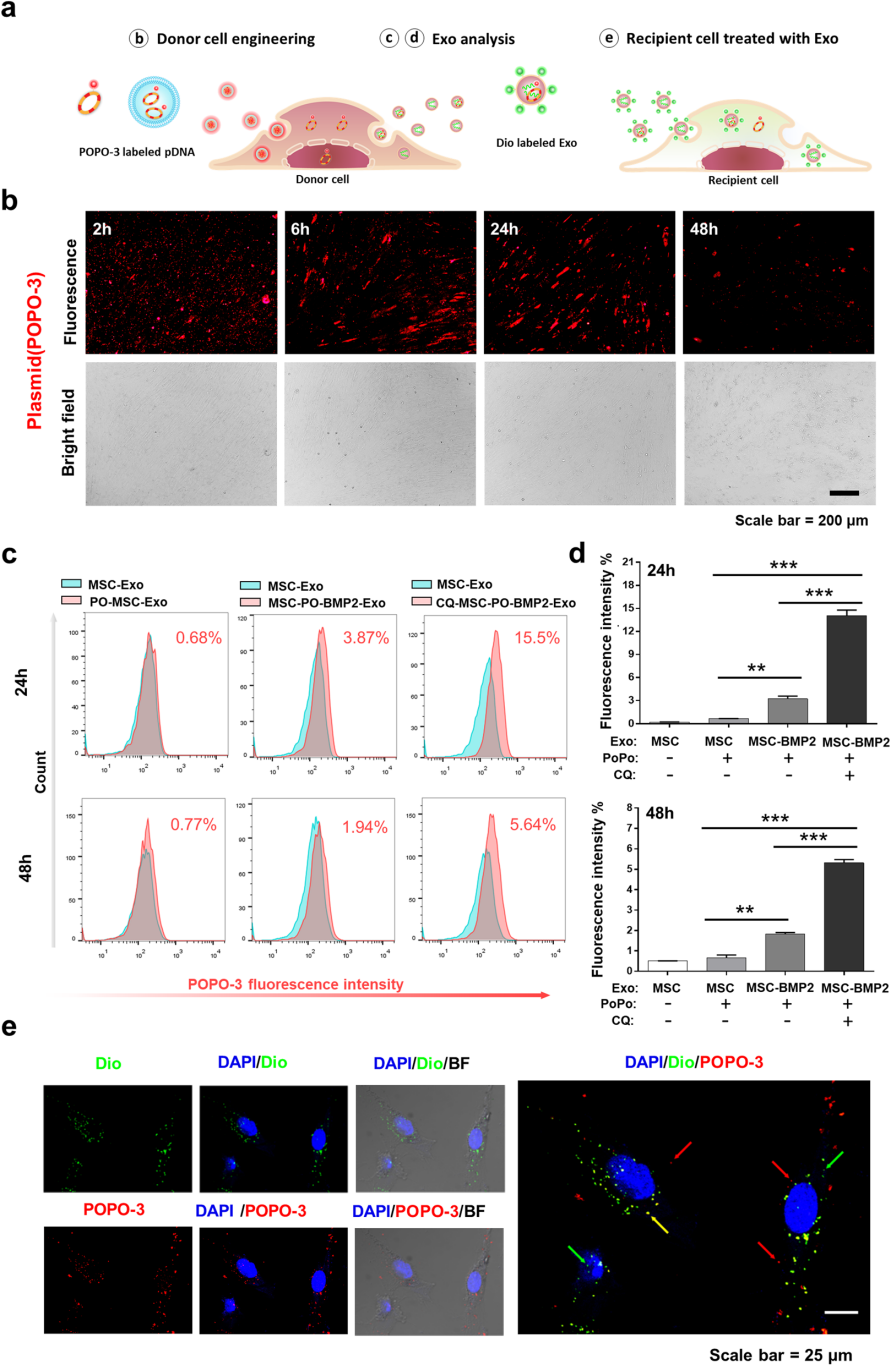
Plasmid DNAs could be packaged by stem cells and transported to recipient cells through exosomes. **a** Plasmid DNAs were labeled with POPO-3 (POPO-pDNA), and observed in donor hMSCs, secreted exosomes and recipient hMSCs. Exosomes were labeled with Dio green fluorescent dye. **b** Visualization of POPO-pDNA in hMSCs during liposome mediated transfection under fluorescent microscopy at 2, 6, 24, and 48 h. Scale bar: 200 μm. **c** Exosomes were collected from culture medium after transfection (PO-MSC-BMP2-Exo) and performed flow cytometry to analyze the amount of POPO-pDNA in exosomes. MSC-Exo was analyzed for baseline, and MSC-Exo containing free POPO-3 iodide was used as control (PO-MSC-Exo). Chloroquine was added in culture medium to inhibit lysosome dependent degradation (CQ-MSC-PO-BMP2-Exo). **d** Quantitative analysis of ratios of exosomes carrying POPO-pDNAs as determined by flow cytometry, n = 3. **e** PO-MSC-BMP2-Exo were then incubated with recipient hMSCs and observed by confocal microscopy. Exosomes were labelled by Dio (Green) and cell nucleus was stained by DAPI (Blue). Some exosomes carrying plasmid DNAs (exhibited yellow color) were found to accumulate around the nucleus (yellow arrows). Green arrow: exosomes, red arrow: plasmid DNA, yellow arrow: exosomes carrying plasmid DNA. Scale bar: 25 μm. **p < 0.01, ***p < 0.001

The osteogenic promoting effects of MSC-BMP2-Exo were proven both in vitro and in vivo*.* Genetically engineered stem cells by liposome-mediated *BMP2* gene delivery altered the nucleic acid content of secreted exosomes. Given that both the liposome and exosomes rely on endosome-mediated cellular transportation, we investigated whether the plasmid DNA can be packaged into the exosomes after transfection. Therefore, the trafficking of plasmid DNA was investigated by labeling them with red fluorescent POPO-3 iodide (Fig. 9a). After hMSC transfection, intense red fluorescence was observed in the hMSCs, indicating that the plasmid DNAs were successfully delivered into these cells by the liposome (Fig. 9b). MSC-BMP2-Exos were then harvested at 24 and 48 h after transfection. The presence of red fluorescence in the exosomes (MSC-PO-BMP2-Exo) was checked by flow cytometry using aldehyde/sulfate latex beads, and 3.87% and 1.94% fluorescence were observed at 24 and 48 h post-transfection, respectively (Fig. 9c). MSC-Exos without red fluorescence were used as the control. POPO-3 iodide was mixed with MSC-Exo and then purified (PO-MSC-Exo). This purified mixture barely showed fluorescence. Interestingly, inhibition of the lysosome-dependent degradation by using chloroquine significantly increased the amount of plasmid DNA in exosomes (CQ-MSC-PO-BMP2-Exo) to 15.5% and 5.64% at 24 and 48 h post-transfection, respectively (Fig. 9c, d). Finally, MSC-BMP2-Exos were further stained with Dio and incubated with recipient cells. The internalization of exosomes in recipient cells could be observed as green fluorescence. Interestingly, confocal microscopy images revealed that plasmid DNA carried by exosomes (yellow fluorescence) could also be observed in recipient cells (Fig. 9e). These results indicated that the plasmid DNA delivered by liposomes could be packaged into secreted exosomes by stem cells during genetic engineering. This method might be useful in fabricating DNA/exosomes complex for clinical applications.

## Discussion

Stem cell-based therapies are believed to have huge therapeutic potential for degenerative diseases, autoimmune and genetic disorders, and tissue/organ injuries, as demonstrated in completed and ongoing clinical studies [35]. This feature is due to their regeneration potency, paracrine activities, inflammation modulation effects, and so on [36]. However, clinical applications of stem cell transplantation raise ethical concerns regarding human embryonic stem cell (hESC) and induced pluripotent stem cells (iPSCs), as well as safety issues, including unwanted differentiation, which induce tumor formation and promote metastasis [37]. On the other hand, administration of exosomes for curing disease, or the so-called “cell-free” therapy, is another strategy to utilize the paracrine and epigenetic modulation effects of stem cells without actually injecting cells into subjects, which avoids tumor formation or other undesired behaviors of cells [7]. As a natural means of bioactive molecule transportation and cellular communication, exosomes exhibit excellent biocompatibility and bioavailability [38]. Their extremely small size facilitates tissue penetration and cellular uptake, making exosomes highly efficient delivery devices for therapeutic components. Furthermore, exosome mediated cell cross-talk is an important method for microenvironment modulation [39].

Extensive efforts have been made to enhance the designed therapeutic effects of exosomes to meet clinical requirements by inserting biocues or producing exosomes mimics [40, 41]. However, technological challenges must still be tackled to increase production efficiency, while maintaining the outstanding biological features of exosomes as much as possible. Gene therapies are designed to modulate gene expressions for preventing, halting, or reversing pathological processes [42]. However, the clinical applications of gene therapy have been hampered by the dilemma between safety and efficiency. We believe that the current problems that hamper the clinical applications of exosomes and gene therapy can be solved by combining these two technologies. To demonstrate this concept, we adapted a stem cell-mediated gene therapy (MSC-GT) strategy and applied it in bone regeneration, in which *BMP2* genetically modified MSCs function as cellular factories. These factories effectively produce exosomes with designed and enhanced therapeutic effects, while increasing the safety compared with that in conventional gene delivery vehicles.

**Fig. 10:**
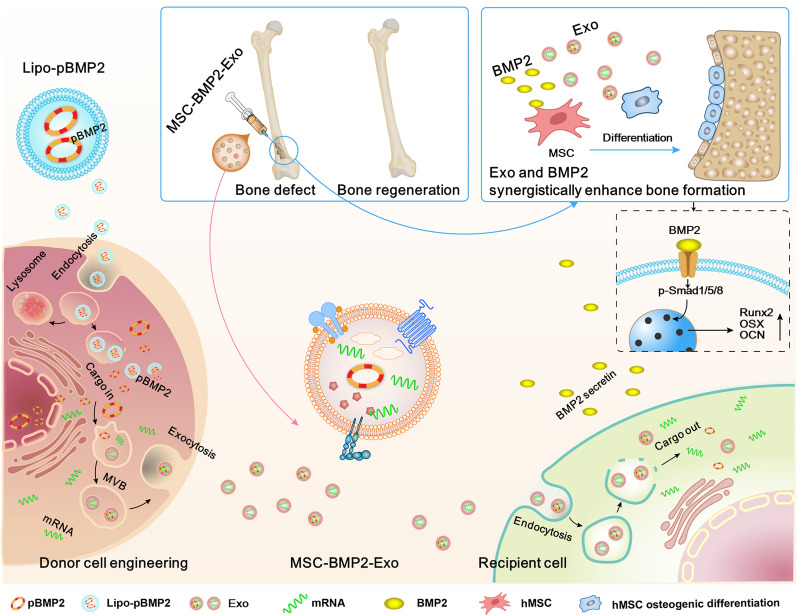
Schematic illustration of exosomes derived from *BMP2* genetically engineered MSCs promote bone regeneration. MSCs were genetically engineered by liposome mediated *BMP2* gene delivery, and altered the content of secreted exosomes. This MSC-BMP2-Exo with the content derived from MSCs and up-regulated osteogenic related gene expression synergistically promoted bone regeneration. Both the liposome and exosomes rely on endosome-mediated cellular transportation, therefore, the pBMP2 delivered by liposome could be re-encapsulated in exosomes to present a safety carrier. This provide a strategy of making use of mediator cells as cellular factories to produce exosomes with designed therapeutic information

BMP2 has been widely used in clinics for the treatment of bone fracture [43, 44]. Gene therapy provides a powerful technology for prolonged delivery of BMP2 over stable periods of time to overcome the short half-lives of protein treatment [45]. It has been well documented that *BMP2* gene transfection could enhance osteogenic effect of MSCs [46]. Direct exosomal engineering with *BMP2* plasmid and utilizing viral vector for engineering exosomal mediator stem cells have demonstrated the enhanced osteogenic effect of exosomes [22]. In this study, mesenchymal stem cells were genetically engineered by liposome-mediated *BMP2* gene delivery to produce exosomes (MSC-BMP2-Exo) with enhanced bone regeneration potency. This effect may be attributed to the synergistic effect of the content derived from MSCs and the up-regulated *BMP2* gene expression (Fig. 10). Specifically, *BMP2* gene transfection enhanced *BMP2* gene expression and up-regulated osteogenic-related mRNA expression in mediator stem cells (Fig. 1). The altered cytoplasmic content of hMSCs further influenced the cargos carried by the secreted MSC-BMP2-Exo, as demonstrated by the up-regulated osteogenic lineage-related mRNAs in exosomes (Fig. 2). This genetic engineering did not affect the morphology, size, markers, and cellular internalization of exosomes (Figs. 2, 3). The mRNAs and plasmid DNAs in cytoplasm are actively identified and segregated on microdomains of limiting membrane of multivesicular bodies (MVBs), and subsequently form intraluminal vesicles (ILVs) [38], the precursors of exosomes. This cargo sorting process involves multiple pathways, but knowledge about the mechanisms is still limited. Cells are likely to sort mRNAs into exosomes by identifying certain motifs in 3’-untranslated regions (3’-UTR) of mRNAs [47]. However, more studies are required to reveal the whole mechanisms. The investigation of plasmid DNAs sorting mechanisms is still at infant stage, and more studies are desired.

As a natural means for cellular communication, exosomes can transport their contents to recipient cells to regulate their functions and behaviors [38]. After interaction with the membrane of recipient cells, exosomes were internalized through endocytic pathway and reached MVBs [38]. A part of exosomes may escape lysosomal digestion by fusing with the membrane of the MVBs, thereby releasing their contents into the cytoplasm of the recipient cells (Fig. 10) Activating surface receptors and signaling or direct fusion with membrane were also proposed as the ways for their regulating effect [11]. This process is still poorly understood, but most importantly the release of exosomal content into the cytoplasm initiated the osteogenesis. The enhanced effects of MSC-BMP2-Exo in osteogenesis were proven by in vitro and in vivo studies through activating BMP2/Smad signaling pathway (Figs. 4, 5, 6, 7). The exosomal content derived from stem cells may also function in regulating recipient cells. Moreover, the healing rate of bone defects is depending upon the treatment strategy [48]. The dosage and treating times of MSC-BMP2-Exo would influence the bone healing time. Future studies are required to optimize the performance for clinic translation.

Exosomes derived from certain types of MSC can enhance bone regeneration [49]. However, the regeneration potencies of exosomes varied among different cell sources, which would be troublesome for mass production and clinical applications. Therefore, technologies that can produce exosomes with enhanced therapeutic effects in an effective and repeatable manner are of great importance for promoting the clinical applications of exosomes. Based on in vitro and in vivo characterizations, we proved that transfecting the mediator MSCs with the *BMP2* gene was a desired strategy. Therefore, this strategy can tremendously broaden sources of exosome donor MSCs. In addition, plasmid DNAs only induce temporary gene expression modulation, which avoids the risks of insertional mutation. It functions around a week, and loading engineered exosomes in hydrogel or scaffold would further prolong the effecting time.

The retention and distribution of exosomes was investigated after local and systemic administrations. Locally injected exosomes could stay at the injection site longer than 48 h, which facilitated in maintaining the local dosage and therapeutic effects. Studies have shown that MSCs derived exosomes have the homing ability to the injured tissue [1, 33]. Interestingly, we also found that after intravenous injection, MSC-BMP2-Exos were found to home to the prepared bone defects (Fig. 8f). This finding suggests that non-invasive administration of MSC-BMP2-Exo might be a potential cure for micro-fractures of bones, which deserves further investigation. Furthermore, exosomes are compatible with the host immune system and have the innate ability to cross biological barriers in vivo [11]. Further studies in exploring the homing effect of exosomes to other injured tissue would promote their application in wound healing the tissue regeneration. We believe that by optimizing the combinations of mediator cells and gene sequences for MSC-GT, many diseases can find corresponding therapeutic exosomes.

Delivery vehicles are important for the effectiveness of gene therapy. Non-viral based gene delivery vehicles, including liposomes and polymeric vectors [50–52], have the advantages of safety and controllable duration of gene expression. However, clinical translation of these systems faces two distinct issues: cytotoxicity of materials and rapid clearance by the mononuclear phagocyte system (MPS) [29], which have not yet been settled. Exosomes, as native intercellular transporters with diverse compositions, protect internal cargos from degradation and prolong systemic retention with minimum adverse effects [11, 28]. Interestingly, our study showed that even though the dosage-dependent toxicity of liposomes was inevitable, the toxic effects could be confined to mediator MSCs, or the so-called cellular factories, by MSCs-mediated gene therapy strategies, whereas exosomes produced by these MSCs exhibited excellent biocompatibility (Fig. 3d).

There have been debating on whether natural exosomes contain DNA or DNA fragments [25, 53]. The contradicting results might be due to the diverse cellular behaviors exhibited by different cell types and difficulties in distinguishing exosomes from other extracellular vesicles or fractions secreted by cells. In this study, we proved that a proportion of plasmid DNAs were re-encapsulated by mediator MSCs and delivered to the recipient cells through exosomes (Fig. 9c–e). In the classical theory of liposome-mediated gene delivery, free nucleic acids are released into the cytoplasm after endosomal escape by fusing with the endosomal membrane [54], by disrupting the endosomal membrane [55], or by fusing with the plasma membrane before entering the nucleus [56]. Meanwhile, cells actively and selectively sort components in the cytoplasm to MVBs. Although the process of how specific cells identify these components remains unclear, our study demonstrated that MSCs could sort plasmid DNAs presented in the cytoplasm and expel them through exosome secretion. Interestingly, this mechanism functions as a re-encapsulation process, through which plasmid DNAs are equipped with a more biocompatible exosomal vehicle (Fig. 10). MVBs have two destinations within cells: merging with cell membranes to release exosomes or merging with lysosomes for degradation [57]. We found that by lysosomal inhibition, the yield of exosomes containing plasmid DNA could be significantly increased. This phenomenon might be applied in the mass production of plasmid DNA/exosome complexes, as substitutional vehicles for gene therapies.

This study developed a strategy of MSC-GT through producing exosomes with designed therapeutic effects. By selecting the proper combinations of transfection tools, gene sequences, and mediator stem cells, this strategy might find applications in the treatments of many diseases. It provides advantages of biocompatibility and bioavailability which may overcome the limitations confronting stem cell therapy, gene therapy and nanomedicine. Moreover, exosomes as nature nanocarriers with intriguing endogenous biofunctionalities and versatile biocomponents triggered the concept of establishing next-generation nanobiotechnology [58, 59]. However, the heterogeneity in living organisms and ambiguity of interacting mechanism with complicated in vivo milieu are the main obstacles impeding their clinical translations [60]. Future progress in isolation technologies and elucidating biological functionalities of exosome-based nanoplatforms would made it possible to envisage highly promising therapeutic applications.

## Conclusions

In the present study, a stem cell-mediated gene therapy strategy is developed to produce exosomes (MSC-BMP2-Exo) with enhanced bone regeneration potency. Mesenchymal stem cells were genetically engineered by bone morphogenetic protein-2 gene to alter the content of the secreted exosomes. The enhanced bone regeneration effect is attributed to the synergistic effect of the content derived from MSCs and the up-regulated *BMP2* expression. The MSC-BMP2-Exos also present biocompatibility, homing ability to the injured site, and plasmid DNA delivering. The accelerating effect in bone healing and the good biocompatibility suggest the potential clinical application of this strategy.

## Material and methods

### Animals

Male C57BL/6 mice (8 weeks old, 25 g) were purchased from Guangdong Medical Laboratory Animal Center (Guangdong, China). BALB/c nude mice (8 weeks old, 20 g) were obtained from Beijing Vital River Laboratory (Beijing, China). Mice were housed with access to water and food.

### Cell culture

Human fetal bone marrow derived mesenchymal stem cells (hMSCs) were purchased from Cyagen Biosciences (HUXMA-01001, USA). Cells between passages three and ten were used and propagated in α-MEM (Gibco, USA) supplemented with 20% fetal bovine serum (FBS, Gibco, USA) and 1% penicillin–streptomycin (Gibco, USA). Mouse hepatocytes were isolated from 8 weeks old male C57 mice, and cultured in L-DMEM (Hyclone, USA) containing 10% FBS and 2% penicillin–streptomycin [61]. Cells were maintained in a humidified atmosphere containing 95% air and 5% CO_2_ at 37 °C.

### Cell transfection

Plasmid pIRES2-ZsGreen1-hBMP2 (pGFP-BMP2) encoding both *BMP2* and *GFP* was constructed as previously described [62]. Plasmid pCI-neo-BMP2 (pBMP2) encoding *BMP2* gene maintained in the lab was used for hMSCs genetically engineering. The hMSCs were transfected with plasmid using Lipofectamine 3000 transfection kit (Invitrogen, USA) according to the manufacturer’s instructions. Briefly, the hMSCs were seeded at a density to ensure 70–90% confluence after a 2-day culture. Equal amounts of Lipofectamin 3000 and P3000 reagent were diluted (1:50 v/v) independently and the plasmid was added into diluted P3000 (1:100 w/v). After incubation, above mentioned reagents were mixed and added to cells. The GFP expression in hMSCs was observed on a fluorescence microscope and quantified by flow cytometry (FACS Aria III, BD Biosciences, USA) 48 h post transfection. For intracellular tracking of plasmid in hMSCs during transfection, the plasmid was labeled with POPO-3 iodide (Invitrogen, USA) and observed under a fluorescence microscope at 2, 6, 24, and 48 h after transfection. For the lysosomal inhibitor assay, the medium was refreshed with α-MEM containing 10% exosome-free FBS (VivaCell, China) and 25 μM chloroquine diphosphate salt (Sigma, USA) 6 h after transfection. The exosomes were extracted at 24 and 48 h after cell transfection and incubated with aldehyde/sulfate latex beads (4 μm in diameter) before performing flow cytometry.

### BMP2 concentration analysis by ELISA assay

After transfection, hMSC supernatants were collected for 48 h and centrifuged at 2000 × g for 20 min to remove cell debris. BMP2 protein levels of transfected and untransfected hMSCs were evaluated by Human BMP2 ELISA kit (Animalunion, China) according to the instructions.

### Exosome isolation

Cells were washed three times with sterile phosphate buffer saline (PBS, Hyclone, USA), and cultured in α-MEM containing 10% exosome-free FBS. After 48 h, the supernatants were harvested and centrifuged at 2000 × g for 30 min to remove cells and debris. The total exosome isolation reagent (TEI, Thermo Scientific, USA) was used to purify exosomes according to the manufacturer's recommendations. To obtain a homogeneous solution, the mixture of above supernatant and reagent (2:1 v/v) was pipetted up and down 30 times and incubated overnight at 4 °C. The mixture was further centrifuged for 1 h at 10 000 × g at 4 °C. The supernatant was then aspirated and discarded, and the exosomes were resuspended with sterile PBS and stored at − 80 °C for further analysis [63].

### Transmission electron microscopy and particle analysis

The exosomes were dropped separately on copper grids, and stained with 2% phosphotungstic acid (pH 7.0, Leagene Biotechnology, China) for 30 s. The staining solution were subsequently removed, and the copper grids loaded with exosomes were air dried overnight. Exosomes were detected using a transmission electron microscope (FEI, Tecnai G2 F20 S-TWIN, USA). For particle size and distribution, exosome suspension was diluted and detected by NTA using Zeta View (Particle Metrix, Germany).

### Zeta potential analysis

MSC-BMP2-Exo or MSC-Exo at 0 day (freshly isolated) and 3 day (stored at − 80 °C) were suspended in PBS at the concentration of 0.5 mg/ml and zeta potential were measured by Zetasizer (NANO ZS, Malvern, UK).

### Western blot analysis

Purified exosome pellets were lysed with RIPA lysis buffer (Beyotime, China) and PMSF (Beyotime, China). The cells were harvested and lysed using whole cell lysis kit (Keygen biotech, China). The protein concentrations were determined using a BCA protein assay kit (Thermo Scientific, USA). After boiling, equal amounts of proteins (20 μg) from different samples were separated by 10% SDS polyacrylamide gels and transferred to a polyvinylidene difluoride (PVDF) membrane (Millipore, USA). The membrane was then blocked with 5% skim milk for 60 min at room temperature and incubated with different primary antibodies overnight at 4 °C, including anti-CD63 (Abcam, USA), anti-CD9 (Abcam, USA), anti-TSG 101 (Abcam, USA), anti-osterix (Abcam, USA), and anti-Runx2 antibodies (Abcam, USA). The PVDF membrane was further incubated with horseradish peroxidase-tagged secondary antibodies separately for 1 h. Protein bands were visualized using enhanced chemiluminescence assay (ECL, Amersham Biosciences, USA) and imaged by the Bio-RAD ChemiDoc gel imaging system (ChemiDoc XRS + system, Bio-Rad, USA).

### Exosome labeling and cellular uptake

3,3’-dioctadecyloxacarbocyanine perchlorate (Dio)was used to label the exosomes (Beyotime, China). Purified exosomes were incubated with Dio (5 mM) for 15 min at 37 °C in the dark and then ultracentrifuged at 120 000 × g for 90 min to remove the unbounded dyes, followed by washing twice with PBS. The labeled exosome pellets were resuspended in PBS prior to use and hMSCs were cultured in a concentration of 1 × 10^5^ cell/mL medium and incubated with Dio-labeled exosomes (80 μg/mL) overnight. DAPI was used to stain the cell nucleus. Intracellular localization of Dio-labeled exosomes was analyzed using a confocal laser scanning microscope (LSM 880, ZEISS, Germany), and quantified by flow cytometry.

### CCK8 assay

Human fetal bone marrow derived mesenchymal stem cells (hMSCs) and hepatocytes were seeded in 96-well plates at a density of 1 × 10^4^ cells per well and allowed to attach overnight. MSC-BMP2-Exo or MSC-Exo (2 μg per well, 100 μl) was incubated with cells for 72 h, and this concentration was adopted according to the reference [34]. For liposome group, the cells were transfected with plasmid-lipid complex (0.1 μg plasmid per well) according to the instructions of Lipofectamine 3000 kit for 6 h and refreshed with culture medium. The cellular viability was detected using a CCK-8 kit assay (Dojindo, Japan) according to the manufacturer’s instructions. Absorbance was measured at 450 nm using a microplate reader (Thermo Scientific, USA). Experiments were repeated at least three times.

### Osteogenic induction of hMSCs

Cells were planted in a 12-well cell culture dish (Corning, USA) at approximately 4 × 10^5^ cell per well. After reaching 70–80% confluence, osteogenic differentiation was induced with the addition of osteogenic medium, consisting of α-MEM medium supplemented with 10% FBS, 1% penicillin streptomycin solution, 50 μg/mL L-ascorbic acid-2-phosphate (Sigma, USA), 10 mM β-glycerophosphate (Sigma, USA), and 10 nM dexamethasone (Sigma, USA). Exosomes were added with 30 μg per well (20 μg/ml) [64] and the osteogenic medium was changed every three days.

### Quantitative real-time PCR

The total RNAs from different kinds of cells were extracted using TRIzol (Invitrogen, USA). The purity and concentration of RNAs were detected by measuring the absorbance on a NanoDrop2000 (Thermo Scientific, USA) at 260 and 280 nm. The samples with ratios from 1.8 to 2.0 were then qualified for the next reverse transcription reaction. An IScript cDNA Synthesis Kit (Thermo Scientific, USA) was used to synthesize cDNAs according to the manufacturer’s instructions. Each real-time PCR was prepared in a 20 μL of reaction mixture and performed on a CFX96 Real-Time system (Bio-rad, USA). Loading control was *β-actin*. PCR primer sequences for gene expression analyses are listed in Table 1. PCR products were proved by melting curve analysis.

**Table 1:** qRT-PCR primer sequences

	Forward primer sequence (5′-3′)	Reverse primer sequence (5′-3′)
*β-actin*	CATGTACGTTGCTATCCAGGC	CTCCTTAATGTCACGCACGAT
*BMP2*	CCGCTCGAGTAAGGCGACATGGTGGCCGGGAC	GACTGGAATTCCTAGCGAAACCCACAACCCT
*Runx2*	CCGCACGACAACCGCACCAT	CGCTCCGGCCCAATCTC
*Osterix*	TAGTGGTTTGGGGTTTTTACCGC	AACCAACTCACTCTTCCCTAAGT
*ALP*	ACGTGGCTAAGAATGTCATC	CTGGTAGGCGATGTCCTTA
*BSP*	GCATCGAAGAGTCAAAATAGAGGAT	TAAATGAGGATAAAAGTAGGCATGCTT
*OPN*	GGACTCCATTGACTCGAACG	TAATCTGGACTGCTTGTGGC

### Alkaline phosphatase and Alizarin red staining

Cells were induced for osteogenic differentiation and supplemented with different exosomes. Alkaline phosphatase was stained using a BCIP/NBT alkaline phosphatase color development kit (Beyotime, China) 2 weeks after culture. The deposition of calcium phosphate was stained with Alizarin Red S (1%, pH 4.2, Solarbio, China) 3 weeks after differentiation. The images were taken by a biological microscope (Olympus, BX53, Japan). Mineralized matrix was quantified by dissolving Alizarin Red S in cetylpyridinium chloride solution (100 mM, Sigma, USA) for 1 h, and read on a multi-plate reader (Multiskan FC Microplate Photometer, Thermo Fisher Scientific, USA) at 562 nm.

### FACS analysis of exosomes

Exosomes were coated onto beads (MW3000, Invitrogen, USA) and FACS analysis was performed. Exosomes (20 μg) were incubated with aldehyde/sulfate latex beads (5 μL, 4 μm in diameter) for 15 min at room temperature. The bead/exosome mixture was then diluted with 1 mL of PBS and incubated for another 2 h at room temperature under gentle shaking. The beads were then spun down for 3 min at 4000 × g, washed with PBS, and resuspended in FACS buffer. The beads were analyzed by flow cytometry using a FACS-Calibur flow cytometer and FlowJo software.

### Mouse femoral defect model and exosome treatment

Bone defects were created at femora to evaluate the bone regeneration effects of exosomes. C57BL/6 male mice were anesthetized with an intraperitoneal injection of 10% chloral hydrate and kept on a warming pad throughout the surgical procedure. The right hindlimb was shaved and aseptically prepared for surgery by disinfecting with 75% ethanol. Skin incisions of approximately 5 mm were made on the right hind limbs from the lateral side, and femora were exposed by splitting the muscle. For the trabecular bone defect model, 1 mm-diameter holes were created at the lateral femoral distal metaphysis using an orthopedic electric drill. The holes were rinsed by injection of saline using a 1 ml syringe to discard bone fragments from the cavity. The incised muscle and skin were closed with nylon sutures. Perforations did not cause significant perioperation or post-operation fractures. Thirty-six mice with femoral trabecular bone defects were divided into three groups. Each group was treated with either 0.9% saline, MSC-Exo, or MSC-BMP2-Exo. Exosomes (50 μg, 1.8 μg/uL) were injected in situ to the bone defect for the first time on the third day after surgery [65], and treated once a week. The same volume of saline was injected as a control group. At each time point, 15 and 30 days after the first exosome treatment, mice were killed by cervical dislocation. Excised femora were fixed in 10% neutral buffered formalin for 24 h at 4 °C in the dark. After 24 h, the samples were rinsed with running water for about 1 h, then transferred into PBS and stored at 4 °C for further micro-CT and immunohistochemical analysis of trabecular bone defect model. For the cortical bone defect model, holes of the same diameter were created at the mid-diaphysis of the femora. Fifteen mice were divided into three groups, and treated with either 0.9% saline, MSC-Exo, or MSC-BMP2-Exo, with five mice in each group. Exosomes were administrated similarly. In vivo micro-CT imaging of cortical bone defect model was performed at 15 and 30 days after the first exosome treatment.

### Micro-CT detection

Mouse femora were separated, and the soft tissue was removed. Bone quality was analyzed using high-resolution micro-CT (SkyScan1176, Belgium). Scanning was performed at a voltage of 60 kV, a current of 417 µA, and a resolution of 9.0 µm/pixel. Software of NRecon, CTAn and μCTVol were used for three-dimensional reconstruction (threshold 71) and parameter analysis. For the cancellous bone defect, 400 slices in the region of distal femur above the growth plate were selected for three-dimensional reconstruction, and a cubical region with 70 slices in the center of defect was chosen for structural parameter analysis. The structural parameter of the trabecular bone, including bone mineral density (BMD), bone volume fraction (BV/TV), trabecular number (Tb.N), and trabecular thickness (Tb.Th), were calculated through model-independent 3D measurement. For the cortical bone defect in the femur, 340 continuous slices and 20 slices with the middle slice in the center of defect were chosen for three-dimensional reconstruction. The isolated cortical region in the defect region with 110 slices was analyzed for BV/TV and cortical thickness (Cr.Th).

### Histological and immunohistochemical analysis

After the micro-CT imaging and analysis were performed, the excised femora were decalcified using 10% EDTA (pH = 7.4) at room temperature for 2 weeks. The samples were dehydrated using a gradient ethanol series and a final xylene step and were subsequently paraffin embedded. Approximately 5-μm-thick sections were made. Sectioning of the paraffin-embedded samples was performed along the longitudinal axis of the femur. Longitudinal sections were prepared using a microtome (Leica, USA) and tungsten carbide blades. Sections were stained with hematoxylin–eosin and saffran (Beyotime, China). Images of the femur defect region were acquired via light microscopy. For immunohistochemical analysis, bone sections were incubated with primary antibodies against anti-phospho Smad1/5/8 (Millipore, USA) overnight at 4 °C, and then covered with secondary antibodies (Servicebio, China) at room temperature for 50 min. After the sections were cleaned in PBS, DAB color developing solution (Servicebio, China) was used for color development. The slices were flushed with tap water to terminate color development. The slices were stained with hematoxylin for approximately 3 min and rinsed with water. Finally, dehydrated seal was performed, and the images were collected and analyzed with a microscope.

### Biodistribution of exoxomes in mice

Exosomes were labeled with Cy5.5 for in vivo fluorescence imaging. Exosomes were incubated with Cy5.5 NHS ester dye (10 mM, Abcam, USA) for 2 h in the dark. The unbound dye was then removed by three washing steps on 100 kDa ultrafiltration tubes (Millipore, USA). The mouse bone defect model was established, as previously mentioned above, on the right distal femur. Exosomes labeled with Cy5.5 (50 μg each leg) were injected in situ at the distal femur on both sides with and without bone defect on the second day after the establishment of the bone defect model. Six nude mice were divided into two groups for MSC-Exo and BMP2-MSC-Exo. In vivo fluorescence images were obtained using an IVIS imaging system (IVIS Spectrum, PerkinElmer, USA). The distribution of Cy5.5-labeled exosomes was observed at 6, 24, or 48 h after injection. The ex vivo fluorescence images were then visualized from the sacrificed mice using the IVIS imaging system. The fluorescence signals were normalized to track the change tendency within a group. To investigate the biodistribution of exosomes in different organs, exosomes labeled with Cy5.5 (100 μg each mouse) were intravenously injected through the tail vein of mice with bone defects on the right distal femur. Mice without bone defects were used for comparison. These mice were sacrificed 48 h after injection, with 3 mice in each group. The heart, lungs, liver, spleen, kidneys and legs were harvested for IVIS imaging.

### Statistical analysis

All experiments were performed in at least three independent cultures/animals per genotype, treatment, and condition. Statistics were assessed using the GraphPad Prism 8 Software and results are presented as mean ± standard deviations (SD). Comparison between two groups was conducted by t-test, whereas multigroup comparisons were conducted by one-way analysis of variance with Tukey’s post hoc test. *P*-values less than 0.05 were considered significant. (*p < 0.05; **p < 0.01; ***p < 0.001).

## Supplementary Information

**Additional file 1: Figure S1**. BMP2 concentration of hMSCs after transfected for 48 h (n=3), *p < 0.05. **Figure S2**. Changes of zeta potential of exosomes at 0 day and 3 day (n=3). **Figure S3**. Cell viability of hepatocytes incubated with exosomes was determined by CCK-8 assay (n=3).

## Data Availability

The data and materials of the study are available from the corresponding author on reasonable request.
