# Supplementary material for: Engineering stem cells to produce exosomes with enhanced bone regeneration effects: an alternative strategy for gene therapy
Source: J Nanobiotechnology. 2022 Mar 15;20:135. doi: 10.1186/s12951-022-01347-3 (PMC8922796; doi:10.1186/s12951-022-01347-3)
Supplement: Supplementary file 1 — Additional file 1: Figure S1. BMP2 concentration of hMSCs after transfected for 48 h (n=3), *p < 0.05. Figure S2. Changes of zeta potential of exosomes at 0 day and 3 day (n=3). Figure S3. Cell viability of hepatocytes incubated with exosomes was determined by CCK-8 assay (n=3). [file 12951_2022_1347_MOESM1_ESM.docx]

**Supplementary Information**

Engineering Stem Cells to Produce Exosomes with Enhanced Bone Regeneration Effects: An Alternative Strategy for Gene Therapy

Feiyang Li ^1†^, Jun Wu ^2,4†^, Daiye Li ^2,4†^, Liuzhi Hao ^1,3†^, Yanqun Li ^1^, Dan Yi ^1^, Kelvin W.K. Yeung ^2,4^, Di Chen ^1^, William W. Lu ^1,4^, Haobo Pan ^1^, Tak Man Wong ^2,4*^, and Xiaoli Zhao ^1,3*^

^1^ Research Center for Human Tissue and Organs Degeneration, Institute of Biomedicine and Biotechnology, Shenzhen Institute of Advanced Technology, Chinese Academy of Sciences, Shenzhen, 518055, China

^2^ Shenzhen Key Laboratory for Innovative Technology in Orthopaedic Trauma, The University of Hong Kong-Shenzhen Hospital, Shenzhen, 518053, China

^3^ University of Chinese Academy of Sciences, Beijing, 100049, China

^4^ Department of Orthopaedics and Traumatology, The University of Hong Kong, Hong Kong, 999077, China

[†] These authors contributed equally to this work.

*Correspondence: zhao.xl@siat.ac.cn; wongtm@hku.hk


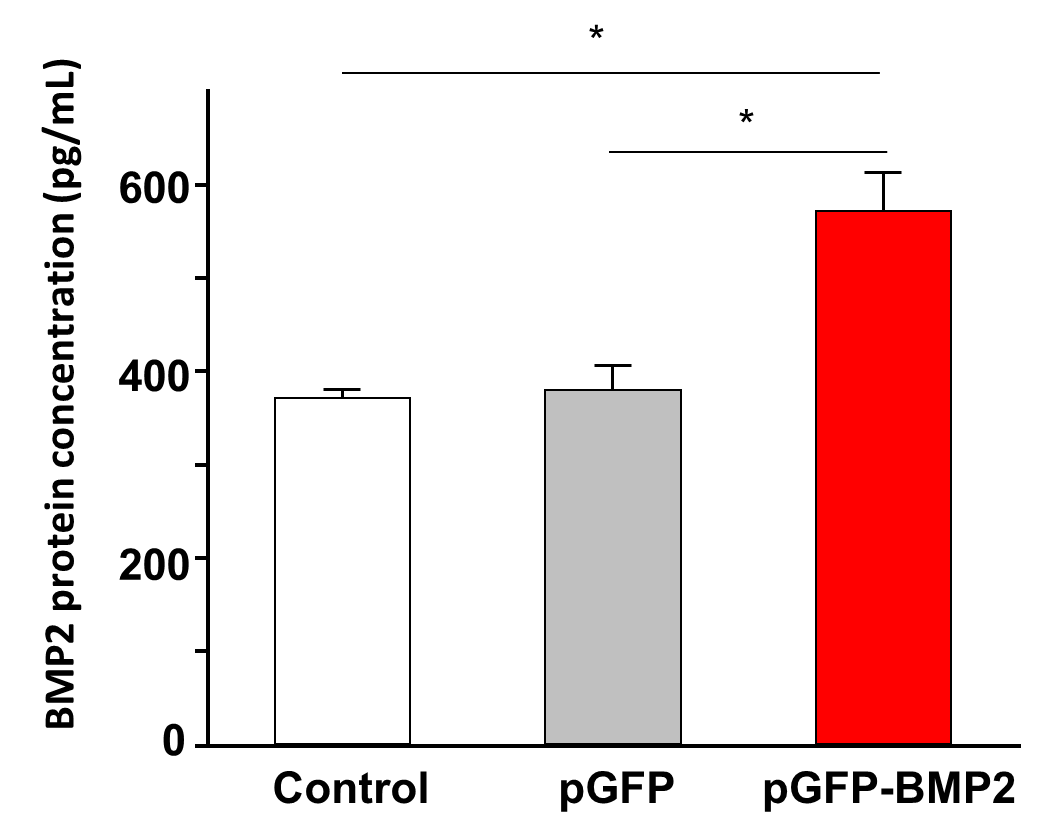


**Figure S1**. BMP2 concentration of hMSCs after transfected for 48 h (n=3), **p* < 0.05


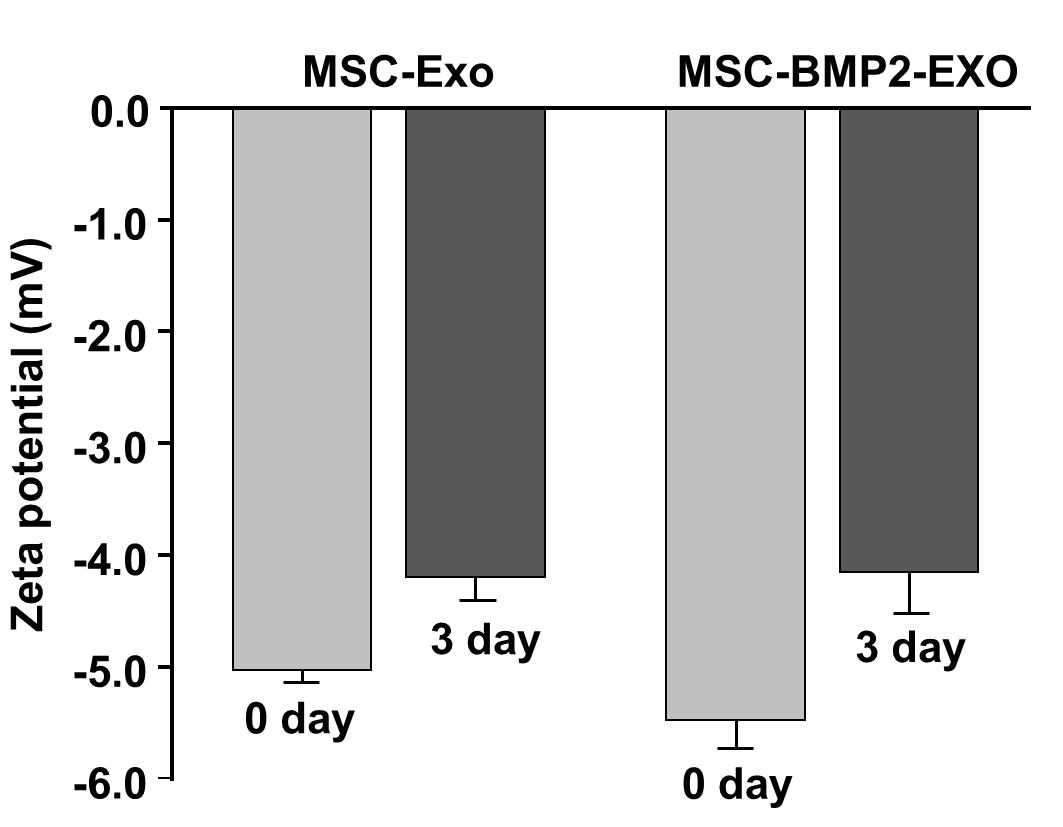


**Figure S2**. Changes of zeta potential of exosomes at 0 day and 3 day (n=3)


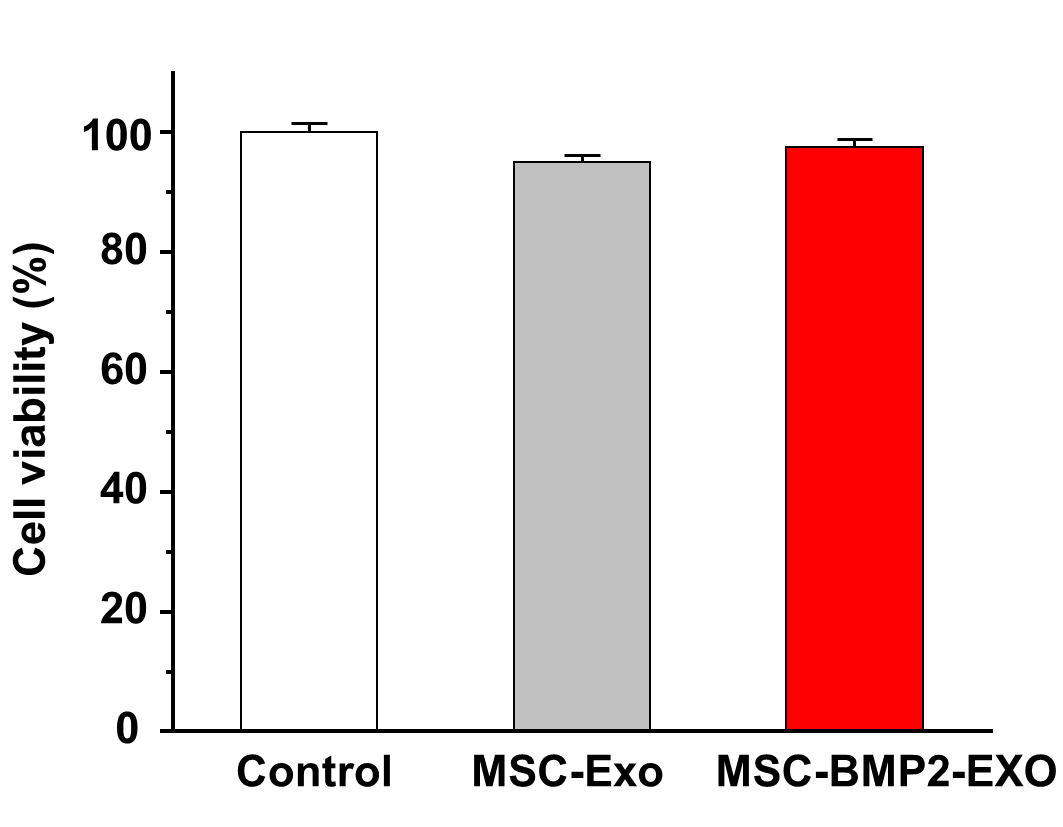


**Figure S3**. Cell viability of hepatocytes incubated with exosomes was determined by CCK-8 assay (n=3)
